# Importance of Work-Related Psychosocial Factors in Exertion Perception Using the Borg Scale Among Workers Subjected to Heavy Physical Work

**DOI:** 10.3389/fpubh.2021.678827

**Published:** 2021-04-29

**Authors:** Emma Sala, Nicola Francesco Lopomo, Cesare Tomasi, Francesco Romagnoli, Alberto Morotti, Pietro Apostoli, Giuseppe De Palma

**Affiliations:** ^1^Unit of Occupational Health, Hygiene, Toxicology and Occupational Prevention, University Hospital Spedali Civili, Brescia, Italy; ^2^Department of Information Engineering, University of Brescia, Brescia, Italy; ^3^Unit of Occupational Health and Industrial Hygiene, Department of Medical and Surgical Specialties, Radiological Sciences and Public Health, University of Brescia, Brescia, Italy

**Keywords:** musculoskeletal disorders, biomechanical overload, risk assessment, Borg scale, psychosocial factors

## Abstract

**Objective:** This study aimed to analyse the role of several environmental and time variables, as well as individual and psychosocial factors, on the perception of exertion, expressed by using the Borg scale, on logistics workers performing heavy manual tasks.

**Materials and Methods:** We enrolled 56 subjects working in logistics sector that were interviewed on the perceived exertion required to execute a task of manual lifting of heavy loads, by using the Borg scale. The interviews were carried out during different shifts, at different times during the shifts and during several different months of the year. We also assessed the workers' anthropometric characteristics, length of service, any musculoskeletal diseases, and physical activity outside work. Workers were also interviewed using the structured OREGE questionnaire, in order to evaluate the main symptoms of stress and work-related psychosocial risk factors.

**Results:** Overall, the subjective perception of the strength exerted by the workers exposed to a high risk of manual handling of loads was moderate. The rating attributed using the Borg scale showed no correlation with any of the investigated variables. 100% of the workers denied to suffer from symptoms of stress, whereas in terms of psychosocial factors, the workload was globally perceived as positive.

**Conclusion:** The study results support the hypothesis that optimal work conditions—from a psychosocial point of view—reduce the subjective perception of exertion by workers even if exposed to a high risk of biomechanical overload.

## Introduction

The main methods that are used in ergonomics to assess the risk of biomechanical overload of the musculoskeletal system rely, in general, on subjective effort perception; the Borg scale is indeed one of the most widespread approach used to estimate workers' strain when they are performing their tasks (1). However, it has been well-documented that further biomechanical overload risk factors—such as dimensions, method and frequency of handling, size of the loads, and posture can affect this perception (2–5).

So far, there are no scientific studies, to the best of our knowledge, investigating the effects of additional variables on the workers' perception of exertion, including the time of work-shift, the period of the year, and the microclimatic conditions at the time of the interview. Furthermore, there are even fewer studies examining the influence of psychosocial risk factors on such subjective perception (6–8). In previous articles, we often addressed the critical issue involved with using scales of subjective evaluation for “exerted force,” as reported by workers or compiled by experts (1, 9, 10). Indeed, we have been widely highlighting the difficulty in assigning risk scores corresponding to the actual level of exertion, when workers are only given a numerical scale with no explanation of what the scores mean. In our experience, the greater the level and the detail of explanation provided for each rating in the scales used for the interview, the fewer subjective overestimations or underestimations are made by workers regarding the risk and the more the risk scores correspond to those assigned by the experts (11, 12).

Within this scenario, we hypothesize that both the subjective context and working environment, as well as the time of the work-shift, may present direct effects on the exertion rating. Therefore, the main aim of this study was to analyse the influence of several environmental and time variables, as well as individual and psychosocial factors, on the perception of exertion, expressed by using the Borg scale, while performing heavy manual tasks, which involve the manual handling of heavy loads during repeated actions.

## Subjects and Methods

This study was performed within the Italian branch of an international enterprise that operates in the logistics sector, in the context of the mandatory periodical health surveillance according to the Italian Legislative Decree 81/2008 and further modifications. All the workers involved in manual load handling expressed their consent to participate to the study, that was performed following the WMA Declaration of Helsinki.

The study sample was represented by 56 male subjects, with an average age of 34 years old (range 20-61 years old). All the main workers' characteristics are summarized in Table 1.

**Table 1:** Characteristics of enrolled workers (no. 56).

**Variables**	**Average ± SD**.	**Min. - Max**.
Age, years	34 ± 10	20-61
Length of service with the company, years	10 ± 7	1-22
Length of service in the role, years	9 ± 6	1-22
BMI, kg/m^2^	24.5 ± 3.0	19-31
Distance from work, km	9.5 ± 7.7	1-32
Borg scale rating	3.7 ± 2.0	0.0-10.0

*BMI, body mass index*.

To define the correct approach for the risk assessment, we preliminary performed an ethnographic on-field analysis, reporting details about the type of manual tasks performed by the workers, the environmental conditions and the planned working shifts.

Accordingly, a risk assessment was carried out into the manual handling of loads, both lifting and pushing-pulling, in accordance with internationally recognized approaches, such as the NIOSH and the psychophysical Snook and Ciriello methods (4, 13). Furthermore, the workers were interviewed on the perceived exertion required to execute a task, by using the Borg scale, which involves assigning a rating ranging between 0 and 10 (1, 14). As previously underlined, while providing the questionnaire we included also the explanations of the numerical ratings, since we reported that interview scales assign much more realistic risk scores if the meaning of said scores are properly explained (10, 11). The interviews were carried out during different shifts, at different times during the shifts (start, middle, and end of shift) and during different months of the year.

We also assessed the workers' anthropometric characteristics [Body Mass Index (BMI)], length of service, any documented musculoskeletal diseases, and lifestyles, with reference to regular physical activity outside work.

Workers also received parts 3 and 4 of the OREGE questionnaire (15). Part 3 (18 questions) investigates the main symptoms of stress, including anxiety (nervousness, tremors, dizziness, vertigo); gastrointestinal disorders; the third stage of stress [sensation of intense fatigue or exhaustion, as described by Selye (16), which reduces the body's ability to adapt to stressful stimuli. Part 4 (26 questions) regards psychosocial factors, including overall and current workload; work pressure; attention and control over work; involvement; immediate social support from superior and colleagues; career prospects (two questions)]. The answers to the questionnaires were evaluated by applying the methodology described by the French INRS (Institut National de la Recherche Scientifique, INRS, 2000) (17).

The statistical analysis was performed using the statistics package SPSS 21.0 (IBM Statistics). Descriptive data analysis, analysis of differences between two (Mann-Whitney analysis) or more groups (Kruskall-Wallis test) and Spearman's correlation analysis were applied. Finally, a factorial variance analysis with normalized Varimax rotation was applied to the psychosocial risk factors.

## Results

Table 1 shows that enrolled workers presented an average length of service with the company of 10 ± 6 years and an average BMI of 24.5 ± 3.0 kg/m^2^; they used to live close to the company (distance between home and work <10 km) and usually travel to work by car. Seven workers reported episodic lower back pain with a diagnosis of disc disease and one worker suffers from chronic epicondylitis. Thirty-two workers (57%) regularly perform exercise at least twice a week.

**Figure 1:**
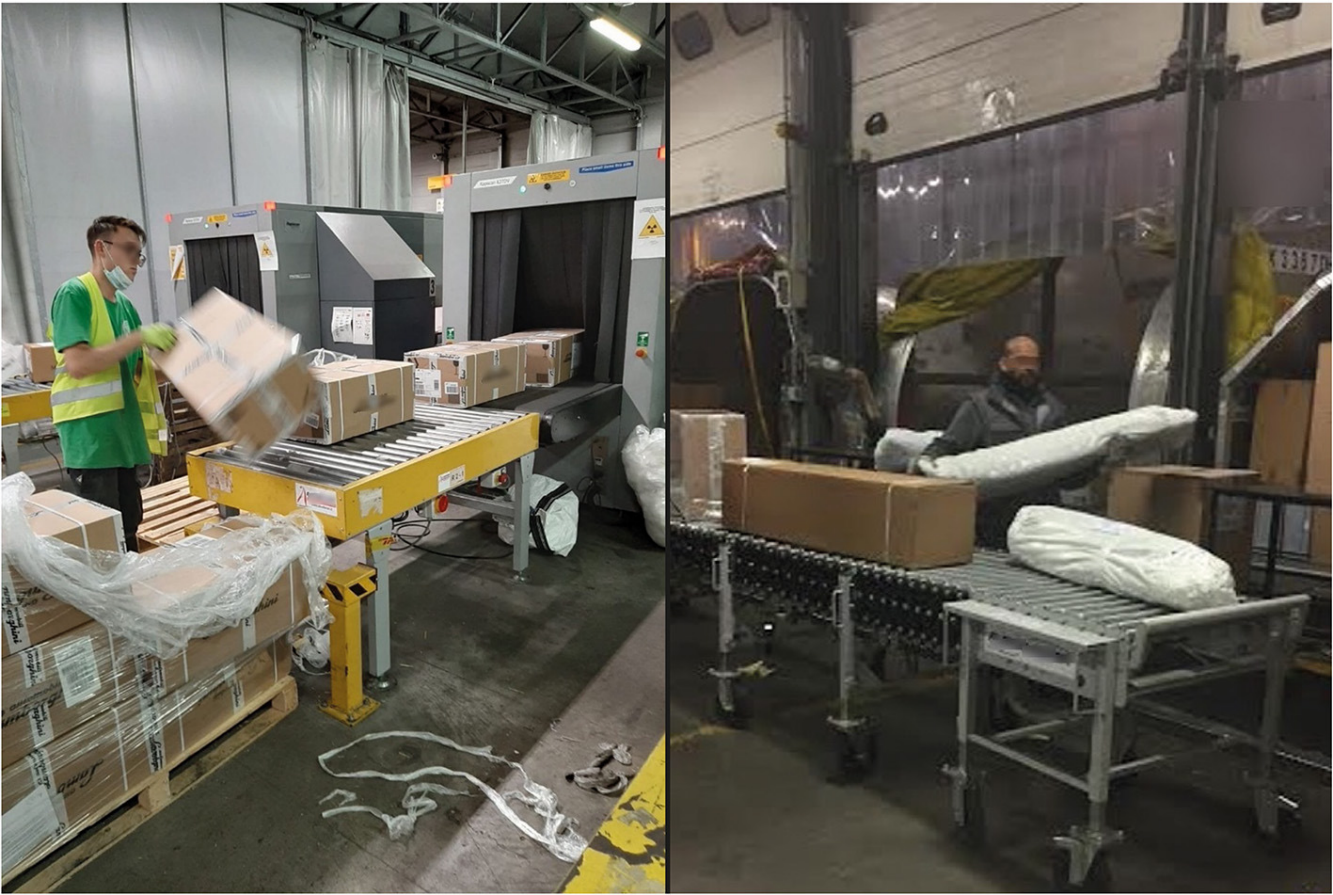
Manual handling of parcels of different weights and dimensions.

**Figure 2:**
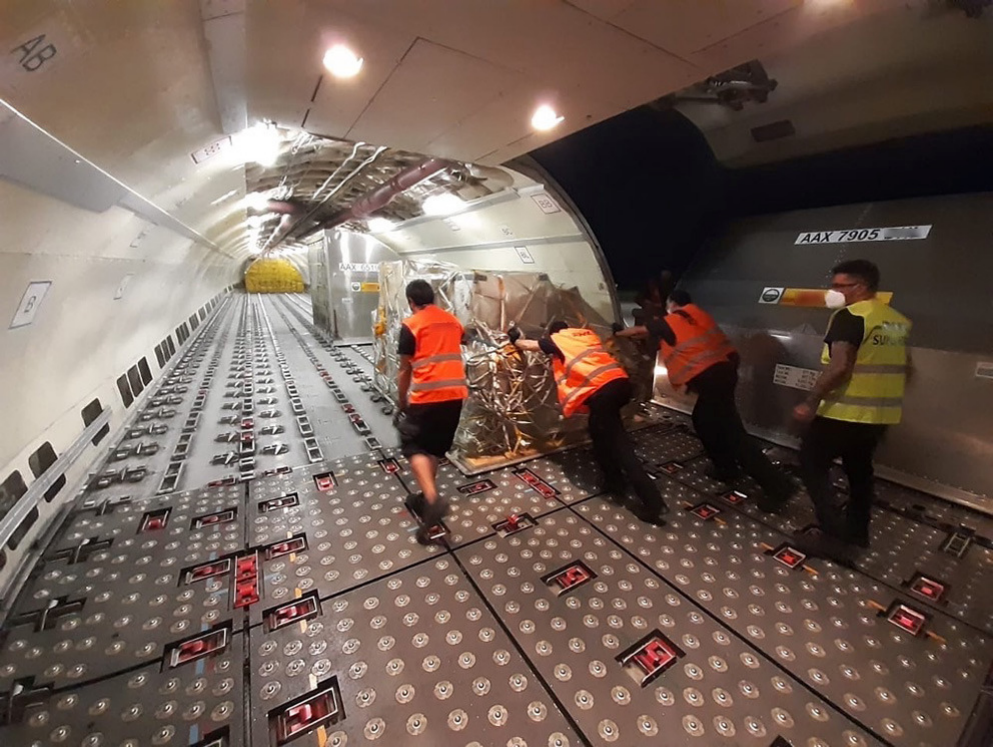
The unit load device (ULD) is pushed by the operators inside the upper deck of a Cargo Aircraft.

From the ethnographic analysis, we reported that warehouse staff were specifically employed to empty and fill incoming and outgoing containers, manually handling packages of varying shapes and sizes (Figure 1). Ramp operators were addressed to push the containers up the ramp and load them into the aircraft (Figure 2). Given the variable nature of the loads to be handled, lifting equipment could not generally be used as aid in these activities; few pneumatic lifting devices were provided only in certain workstations within the warehouse, but these systems could not be widely used due to the package different sizes. The ramp operators always work outdoors, regardless of the weather conditions, while the warehouse staff works in a closed environment with air conditioning, handling loads with greater frequency. The Supervisors (SPVs) coordinate the teams and may provide their support to perform manual tasks, where necessary.

Concerning the working daily planning, work was organized across 27 different types of shifts with different durations (median 7 h; min. 3 h, max. 10 h), with times ranging between 6 p.m. and 12 a.m. and, in most cases, including a number of nocturnal hours (from 0 to 5 h, median 2 h 30 min) defined in accordance with the Italian legislation (Legislative Decree no. 66/2003; work between midnight and 7 a.m.).

The risk assessment into the manual handling of loads as part of lifting and handling operations, as well as pushing-pulling containers, highlighted very high-risk scores (much higher than 3), due to both the weight of the objects being handled and way they are handled, as well as the size of the loads. However, the subjective perception of the strain was not as high, with an average rating of 3.7 ± 2.0 on the Borg scale.

Table 2 shows the breakdown of the ratings obtained in the subgroups of workers classified by their role, areas of prevalent muscular effort, whether or not they suffer from diseases affecting the spine or upper limbs, the time of the interview, the period of the year (hot months vs. cold months) and whether or not they perform regular exercise. There were no cases of statistically significant differences in the perception of exerted force among the different subgroups.

**Table 2:** Distribution of Borg's ratings [median (interquartile range)] in workers classified by occupational, environmental, time, and individual variables.

**Variables (*N*)**	**Borg's score, median (IQR)**
**Role**	Warehouse (*N* = 38)	3.00 (2.38-6.00)
	Ramp (*N* = 18)	3.00 (2.75-4.00)
**Body part under exertion**	Upper limbs (*N* = 9)	3.00 (2.00-3.75)
	Upper limbs > Spine (*N* = 2)	5 (3.00)
	Spine (*N* = 33)	3 (2.25-4.50)
	Spine > Upper limbs (*N* = 12)	4 (3.00-5.50)
**Time of the shift**	First half of shift (*N* = 22)	3 (3.00-6.00)
	Second half of shift (*N* = 21)	3.50 (2.00-4.00)
	Off shift (*N* = 13)	3.00 (2.00-4.00)
**Months**	October-March (*N* = 34)	3 (2.38-4.00)
	April-September (*N* = 22)	3 (2.75-4.25)
**Spine or upper limb diseases**	Yes (*N* = 8)	5.00 (3.00-6.00)
	No (*N* = 48)	3.00 (2.13-4.00)
**Regular exercise**	Yes (*N* = 32)	3.00 (2.25-5.50)
	No (*N* = 24)	3.00 (2.63-4.00)

*There are no statistically significant differences among the different subgroups*.

In addition, the rating attributed using the Borg scale showed only a slight significant positive relationship with duration of the specific job (*R*^2^ 0.07, beta 0.27, *p* < 0.05), but not with working hours/day, worked hours, work-shift, month (season) and time of the interview, BMI and home distance from the workplace (data not shown).

As regards the OREGE questionnaire, 100% of the workers denied suffering from symptoms of stress (Part 3 of the Questionnaire, data not shown). In terms of psychosocial risk factors, the workload was perceived as being demanding both generally speaking (100%) and at the time of the interview (80%); work was judged not pressing (100% of workers); work required attention (100% of workers), control (100% of workers), and workers' involvement (98%); at work there was a good social support from the boss and colleagues (100%), with no concern about future job prospects (100%) (Supplementary Table 1). These results were indeed expected, as they are typical of the investigated sector. In logistics, infact the workers have little organizational influence that depends on sophisticated softwares and management systems, but they have high commitment to work and influence on overall work quality that requires a great professionalism and experience.

The questionnaires on psychosocial factors were therefore evaluated statistically by factorial analysis, excluding the questions about “social support from the boss” and “social support from colleagues,” that gained the same answers across workers. The analysis showed that 37% of the overall variance of psychosocial risk factors was explained by two components, including work pressure and current workload (first component) and professional future and involvement (second component) (Table 3).

**Table 3:** Results of the factorial analysis of psychosocial risk factors.

	**1**	**2**	**3**	**4**
**Work pressure**	0.834			
**Current workload**	0.795			
**Professional future**		0.884		
**Involvement**		−0.532	0.505	
Workload in general			0.846	
Control over work				0.732
Attention				−0.579
Variance (70%)	19%	18%	17%	16%

*37% of the variance is explained by the first four psychosocial factors, in bold*.

## Discussion and Conclusions

The main aim of the study was to verify the interference of individual and environmental factors, including BMI, health status, age, length of service, microclimatic conditions, work-shift, time of the shift, performing regular exercise outside work on the subjective perception of physical exertion while performing a work with a very high risk of biomechanical overload by manually handling loads. Unlike what we expected according to literature data, the study results underline that the exertion perception is not significantly influenced by the job or by any other environmental, time and individual variables taken into consideration (2, 6, 8, 18).

In our opinion, such results should be interpreted largely considering the outcomes obtained in the OREGE questionnaires, in particular the part investigating psychosocial factors. The investigated worker sample can be mainly described as a working population with no stress symptoms, a good company environment, a good salary, career prospects, managers who pay attention to the workers' needs, good horizontal and vertical relationships, confidence in the future of the company and job security. The worker study group was constituted by: university students who preferentially work in the evening/night and can study in the morning/afternoon; moms who usually cover the shift 22:00-01:00, when the children sleep; fathers who work 6 h and are paid as for 8 h. The ramp agents have a very high salary, including several allowances. The company-worker relationship was very good.

Further, the evidence that even the time of the shift when the workers were interviewed had no influence on the obtained results could be interpreted specifically considering the psychosocial factors; more in detail, these subjects gave positive feedback about night shifts, receiving full-time pay for reduced hours (6 h), which enable them to perform further activities during the day (e.g., study, family, second job, etc.). These findings are in agreement with studies highlighting how psychosocial factors can play a decisive role in the perception of disorders and in the assessment of the risk of biomechanical overload of the upper limbs (7, 19–24). On the other hand, psychosocial discomfort can lead to incorrect working practices, which can amplify the effects of risk factors specific to the role, despite not necessarily being significant in themselves (12, 25).

We recognize that our study is affected by main limitations, including (i) its cross-sectional study design making it impossible to determine direction of causation and raising the possibility that the study sample was unrepresentative because of healthy worker selection; (ii) the small sample size and the possibility that relationships were missed because of inadequate statistical power. The results would need confirmation by a different study design, and a larger sample size. According to the experience of the principal investigator, who was the occupational health physician of the company by long time, the obtained results were not affected by the healthy worker effect, as the worker group was stable over years. The obtained results are in agreement with our previous studies, highlighting the role of work-related psychosocial risk factors on the workers' perception of exertion and the extent to which the subjective methods of estimating exertion are affected by these factors in terms of overestimating or underestimating the real risk (10–12). Therefore, a reliable evaluation of exertion should be based on the quantification of objective parameters using several tools able to acquire environmental and physiological information—such as electromyography, dynamometers, load cells, and inertial sensors—combined with ergonomic methodologies of risk assessment (3, 5, 15, 26–30).

In conclusion, in the specific investigated setting, the study allows us to hypothesize that optimal work conditions—from a psychosocial point of view—could be the reason for subjective underestimation of exertion by workers exposed to a high level of risk of biomechanical overload.

We also want to emphasize the importance of proper health surveillance even in optimal situations from the point of view of psychosocial factors. Workers exposed to heavy duty jobs, but “apparently” ignoring its hardness may have clinical problems and even occupational illness, if neglected; but the workers of our study, undergo periodic and accurate health checks by the occupational physician who has assessed the worker's health status for many years with targeted questionnaires on symptoms, objective examination and second level examinations, when required.

## Data Availability Statement

The original contributions presented in the study are included in the article/supplementary material, further inquiries can be directed to the corresponding author/s.

## Author Contributions

ES and GD conceptualized and designed the study and made the decision to submit. ES designed the data collection instrument and administered the questionnaires to the workers. CT did the statistical analyses. ES, NL, FR, AM, and PA analyzed the data and commented on the manuscript. All authors critically revised the paper, approved the final study, and agreed to be accountable for all aspects of the work in ensuring that questions related to the accuracy or integrity of any part of the work are appropriately investigated and resolved.

## Conflict of Interest

The authors declare that the research was conducted in the absence of any commercial or financial relationships that could be construed as a potential conflict of interest. The handling Editor declared a past co-authorship with several of the authors ES, PA, and GD.
