# Supplementary material for: Importance of Work-Related Psychosocial Factors in Exertion Perception Using the Borg Scale Among Workers Subjected to Heavy Physical Work
Source: Front Public Health. 2021 Apr 29;9:678827. doi: 10.3389/fpubh.2021.678827 (PMC8116491; doi:10.3389/fpubh.2021.678827)
Supplement: Supplementary file 1 [file Table_1.DOCX]

**Supplemental Table 1.** Distribution of answers to the OREGE questionnaire, part 4.

| Items | Questions | *No.* | *%* |
| --- | --- | --- | --- |
| *Workload in general* | *Are you forced to work quickly?* |  |  |
|  | *Rarely* | - | - |
|  | *Sometimes* | 5 | 9 |
|  | *Quite often* | 7 | 13 |
|  | *Very often* | 44 | 78 |
|  | *Do you have to guarantee high hyperproductivity?* |  |  |
|  | *Rarely* | - | - |
|  | *Sometimes* | 1 | 1 |
|  | *Quite often* | 2 | 4 |
|  | *Very often* | 53 | 95 |
|  | *Are you generally very busy?* |  |  |
|  | *Rarely* | - | - |
|  | *Sometimes* | - | - |
|  | *Quite often* | 1 | 2 |
|  | *Very often* | 55 | 98 |
| *Current workload* | *In this moment, are you very busy?* |  |  |
|  | *No* | - | - |
|  | *Yes* | 56 | 100 |
|  | *Is the work tied (in this moment)?* |  |  |
|  | *No* | - | - |
|  | *Yes* | 56 | 100 |
|  | *Do you experience delays in completing your work (in this moment)?* |  |  |
|  | *No* | 56 | 100 |
|  | *Yes* | - | - |
| *Work pressure* | *Are you overworked?* |  |  |
|  | *Never* | *56* | *100* |
|  | *Sometimes* | - | - |
|  | *Often* | - | - |
|  | *Always* | - | - |
|  | *Is the work tied (in general)?* |  |  |
|  | *By no means* | - | - |
|  | *A little* | - | - |
|  | *Quite* | 8 | 14 |
|  | *Much* | 48 | 86 |
|  | *Do you experience delays in completing your work (in general)?* |  |  |
|  | *No* | 56 | 100 |
|  | *Yes* | - | - |
|  | *Are there any performance targets (piecework)?* |  |  |
|  | *Never* | 56 | 100 |
|  | *Sometimes* | - | - |
|  | *Often* | - | - |
|  | *Always* | - | - |
| *Attention* | *Does your work require high attention?* |  |  |
|  | *Never* | - | - |
|  | *Sometimes* | - | - |
|  | *Often* | 2 | 4 |
|  | *Always* | 54 | 96 |
|  | *What is the risk of error in your job if you lack concentration for a moment?* |  |  |
|  | *None* | *-* | *-* |
|  | *Little* | *-* | *-* |
|  | *Quite* | 40 | 71 |
|  | *Much* | 16 | 29 |
| *Control over work* | *Can you choose the order in which to carry out your tasks?* |  |  |
|  | *Freely* | - | - |
|  | *Much* | - | - |
|  | *Mildly* | - | - |
|  | *A little* | - | - |
|  | *Very little* | 56 | - |
|  | *Can you decide what amount of work you do?* |  |  |
|  | *Freely* | - | - |
|  | *Much* | - | - |
|  | *Mildly* | - | - |
|  | *A little* | - | - |
|  | *Very little* | 56 | 100 |
|  | *Can you work as you wish?* |  |  |
|  | *Freely* |  |  |
|  | *Much* | 9 | 16 |
|  | *Mildly* | 38 | 68 |
|  | *A little* | 9 | 16 |
|  | *Very little* |  |  |
|  | *What influence do you have on the quality of the work entrusted to you?* |  |  |
|  | *Very big* | 42 | - |
|  | *Much* | 14 | - |
|  | *Mild* | - | - |
|  | *Little* | - | - |
|  | *Very little* | - | - |
|  | *Can you take initiative in your work?* |  |  |
|  | *Often* | - | - |
|  | *Sometimes* | - | - |
|  | *Rarely* | 42 | 75 |
|  | *Never* | 14 | 25 |
| *Participation* | *Have you taken part in decisions regarding your work?* |  |  |
|  | *Always* | - | - |
|  | *Much* | - | - |
|  | *Occasionally* | - | - |
|  | *Little* | 3 | 5 |
|  | *Almost never* | 53 | 95 |
|  | *Do you participate in the organization of your work?* |  |  |
|  | *Always* | 4 | 7 |
|  | *Much* | 52 | 93 |
|  | *Occasionally* | - | - |
|  | *Little* | - | - |
|  | *Almost never* | - | - |
|  | *Do you decide which part of the job you are going to do?* |  |  |
|  | *Always* |  |  |
|  | *Much* |  |  |
|  | *Occasionally* | 2 | 4 |
|  | *Little* | 52 | 92 |
|  | *Almost never* | 2 | 4 |
| *Social support of the boss* | *Do your closest superiors quit their job if needed to help you with yours?* |  |  |
|  | *Much* | 56 | 100 |
|  | *Quite* | - | - |
|  | *A little* | - | - |
|  | *By no means* | - | - |
|  | *Can you rely on your closest superiors in case of difficulty in the job?* |  |  |
|  | *Much* | 56 | 100 |
|  | *Quite* | - | - |
|  | *A little* | - | - |
|  | *By no means* | - | - |
| *Social support of colleagues* | *Do your colleagues leave their job if necessary to help you with yours?* |  |  |
|  | *Much* | 54 | 96 |
|  | *Quite* | 2 | 4 |
|  | *A little* | - | - |
|  | *By no means* | - | - |
|  | *Can you rely on your colleagues in case of difficulty in the job?* |  |  |
|  | *Much* | 54 | 96 |
|  | *Quite* | 2 | 4 |
|  | *A little* | - | - |
|  | *By no means* | - | - |
| *Professional future* | *In your opinion, how likely is your job to be canceled next year?* | - | - |
|  | *By no means* | 46 | 82 |
|  | *A little* | 10 | 18 |
|  | *Quite* | - | - |
|  | *Much* | - | - |
|  | *In your opinion, how likely is your job to be automated next year?* |  |  |
|  | *By no means* | 49 | 87 |
|  | *A little* | 7 | 13 |
|  | *Quite* | - | - |
|  | *Much* | - | - |

**Supplemental Table 2**: Borg scale with examples provided to workers

| **Rating** | **Exertion** | **Exertion description** |
| --- | --- | --- |
| 0 | None at all | No exertion at all |
| 0.5 | Extremely light | Light effort (typing on a computer keyboard) |
| 1 | Very light | Light and easily bearable effort (moving a magazine) |
| 2 | Light | Modest but perceptible effort (but bearable) |
| 3 | Modest | Perceptible effort (no need to stop) |
| 4 | Modest (+) | Perceptible and demanding effort (need to stop every now and then) |
| 5 | Hard | Very demanding effort (need to stop frequently) |
| 6 | Hard (+) | The exertion alters your facial expression |
| 7 | Very hard | You need to use your shoulders and torso to manage the exertion |
| 8 | Very hard (+) | The exertion is so intense that it is almost unbearable |
| 9 | Very hard (++) | The exertion is so intense that it can only be tolerated for a few minutes at a time |
| 10 | Maximum | Exhausting exertion (full force required) |
